# Supplementary figures and images for: Wasl is crucial to maintain microglial core activities during glioblastoma initiation stages
Source: Glia. 2022 Feb 22;70(6):1027–51. doi: 10.1002/glia.24154 (PMC9306864; doi:10.1002/glia.24154)

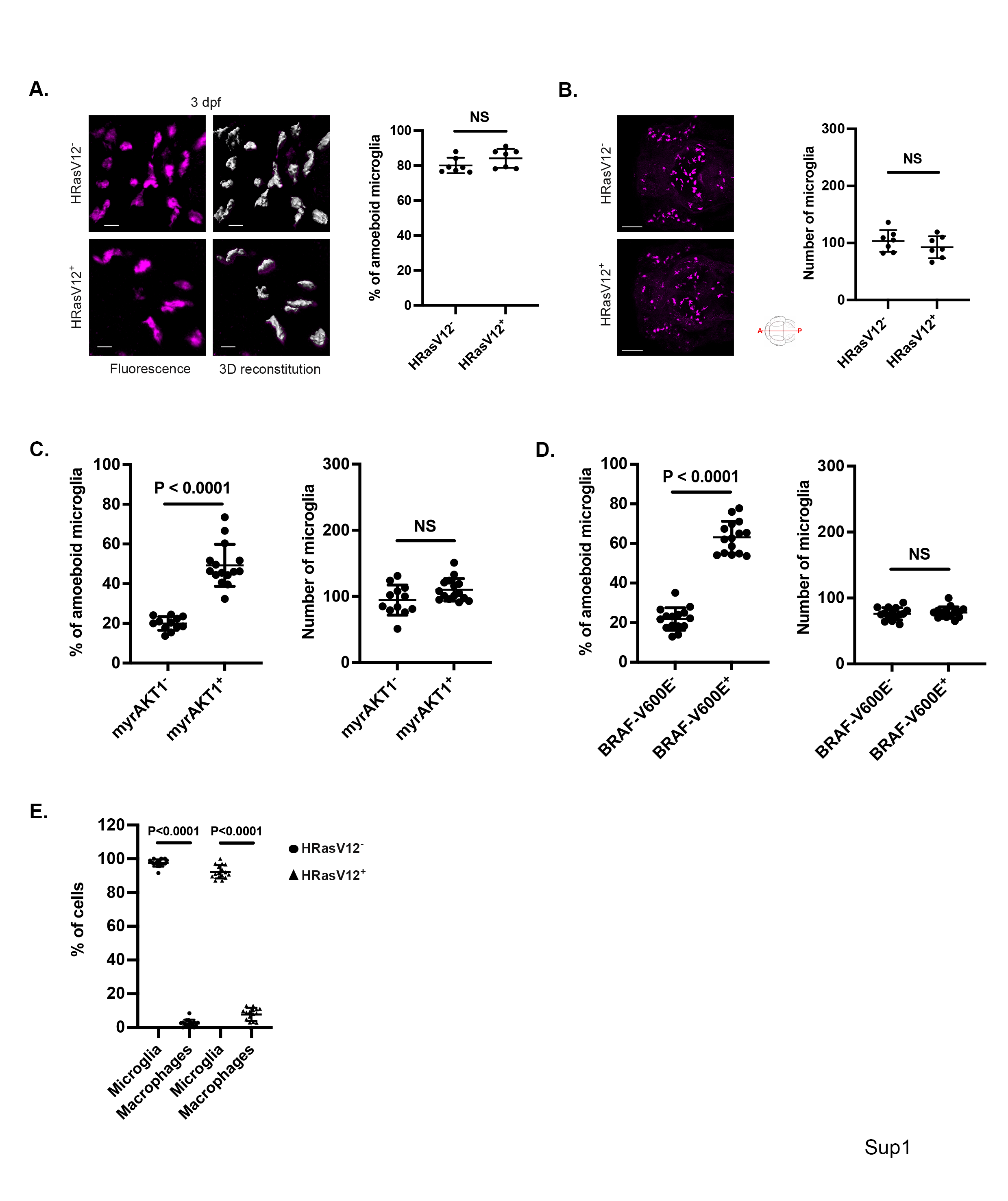

Supplement: Supplementary file 1 — Figure S1: Sup1: HRasV12 expression in the proliferating domains of the developing CNS doesn't alter microglia morphology and number at 3 dpf. (A) Close‐ups of microglia from 3 dpf HRasV12− (top panels) and HRasV12+ (bottom panels) larvae (left panels) and their segmented images in 3D (right panels) using Imaris surface tool, to assess microglia morphology. Scale bar represents 10 μm. The number of amoeboid microglia was quantified within the microglial population of 3 dpf control and HRasV12+ larvae. Results are expressed as a percentage of total microglia. HRasV12‐: n = 7; HRasV12+: n = 7; N = 3. Error bars represent mean ± SD. (B) Confocal images of microglia population (magenta) of 3 dpf HRasV12‐ (top panel) and HRasV12+ (lower panel) brains. Scale bar represents 100 μm. The number of microglia from 3 dpf HRasV12‐ and HRasV12+ brains was quantified. HRasV12‐: n = 7; HRasV12+: n = 7; N = 3. (C‐D) The percentage of amoeboid microglia and the total number of microglia from 5 dpf myrAKT1‐, myrAKT1+, BRAFV‐600E‐ and BRAF‐V600E+ brains were quantified. myrAKT1‐: n = 15; myrAKT1+: n = 15; BRAF‐V600E‐: n = 15; BRAF‐V600E+: n = 15; N = 2. (E) The number of microglia (GFP+;4C4+) and macrophages (GFP+;4C4‐) was quantified within the myeloid cell population of 5 dpf control and HRasV12+ larvae. Results are expressed as a percentage of total myeloid cells. HRasV12‐: n = 15; HRasV12+: n = 15; N = 2. Error bars represent mean ± SD. Images were captured using a Zeiss LSM880 confocal microscope with a 20X/NA 0.8 objective. All images represent the maximum intensity projections of Z stacks. [file GLIA-70-1027-s001.tif]

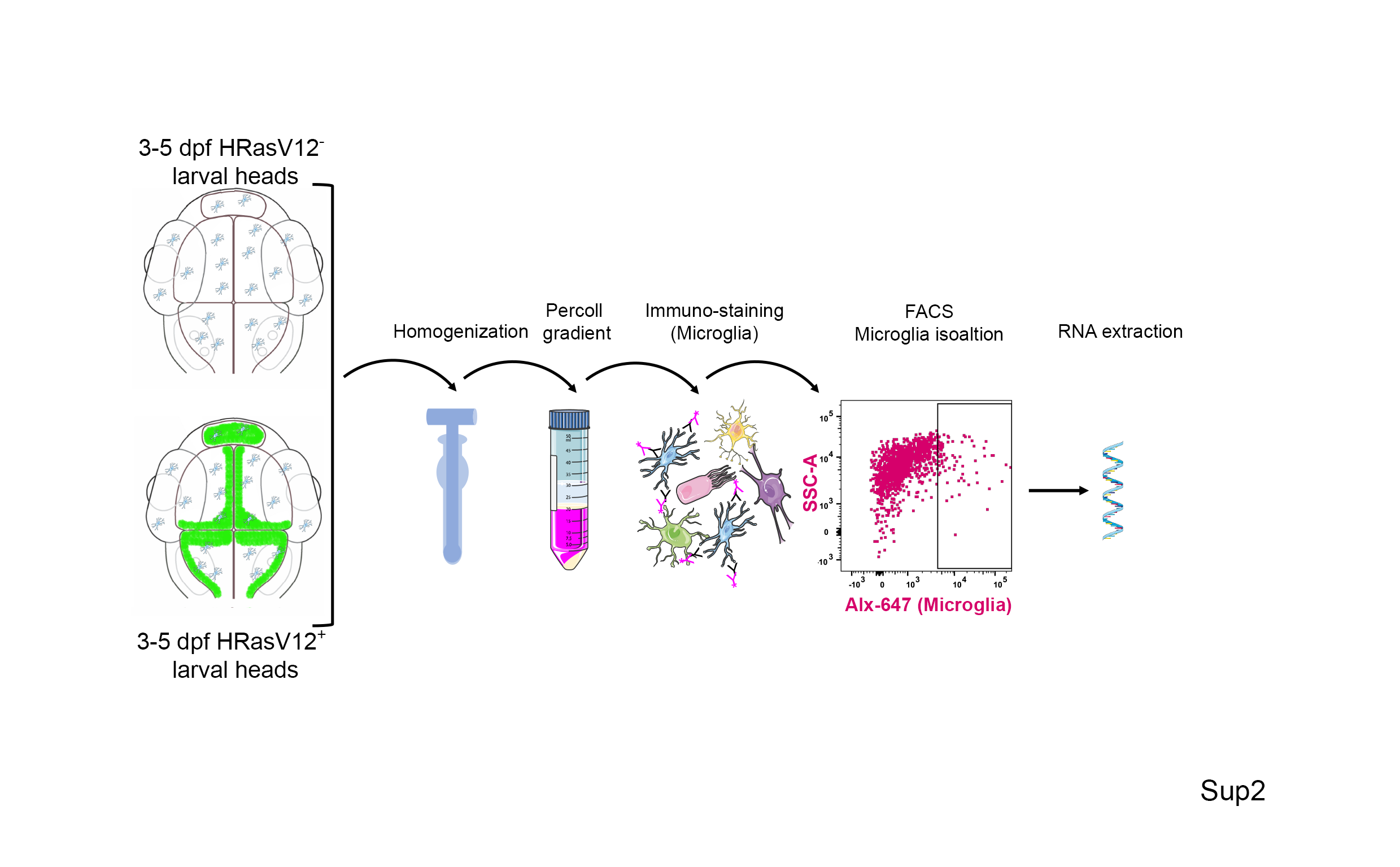

Supplement: Supplementary file 2 — Figure S2: Sup2: Protocol of microglia isolation from HRasV12 ‐ and HRasV12 + larvae. Schematic representation of the protocol used to isolate 4C4+ microglia from larval zebrafish brains of 3 and 5 dpf HRasV12‐ and HRasV12+ larvae to perform RNA extraction. [file GLIA-70-1027-s008.tif]

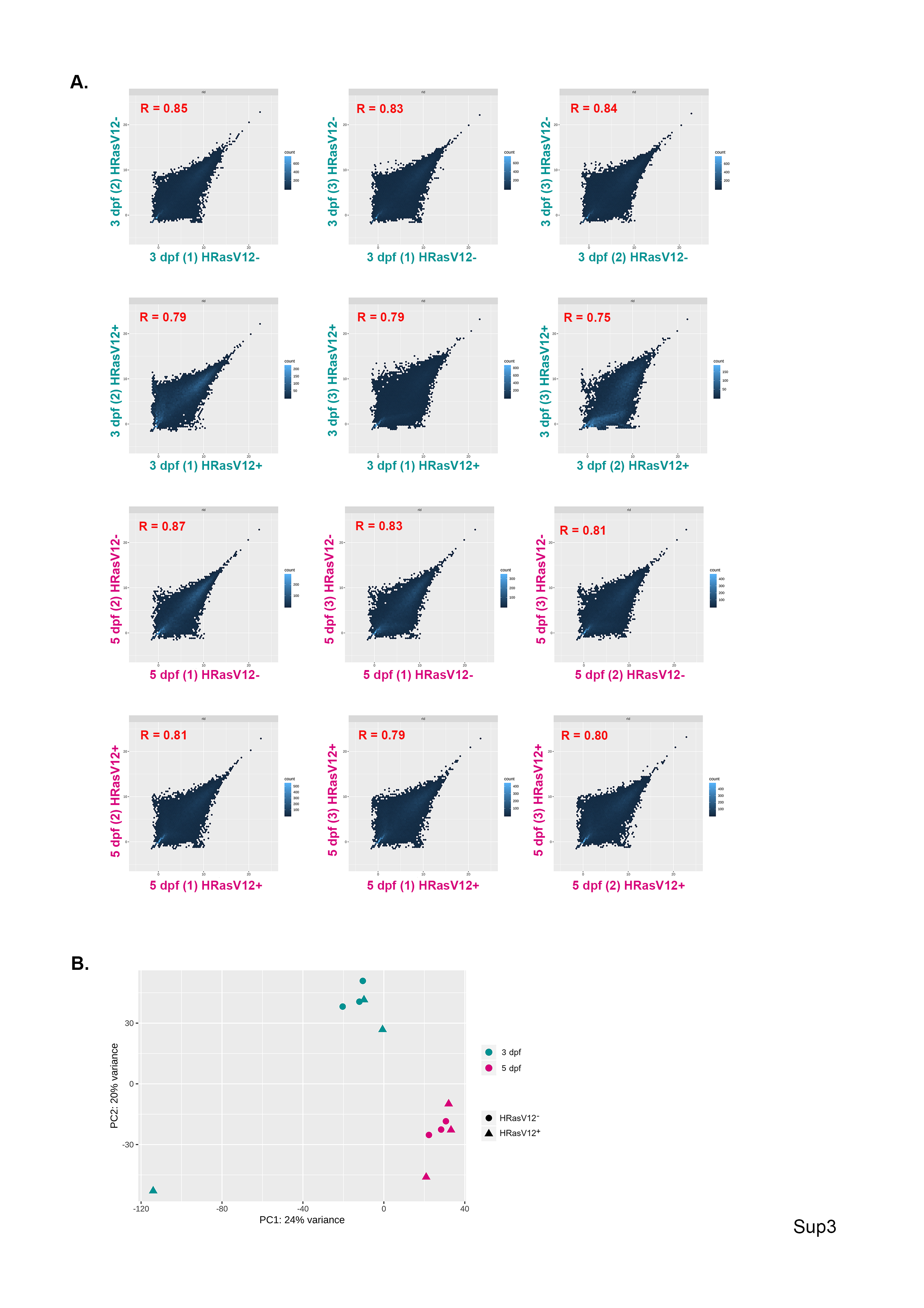

Supplement: Supplementary file 3 — Figure S3: Sup3: Correlation between biological replicates of isolated microglia from HRasV12 ‐ and HRasV12 + brains. (A) Normalised counts from 3 dpf replicates 1 and 2, and 1, and 3 HRasV12‐ and HRasV12+ (green). Normalised counts from 5 dpf replicates 1 and 2, and 1, and 3 HRasV12‐ and HRasV12+ (magenta). Pearson's r is indicated. Colors represent point density (Dark blue: low; light blue: high). (B) Principal component analysis (PCA) score plot obtained from normalised counts of isolated microglia from 600 HRasV12‐ (●) and HRasV12+ (▲) larval brains at 3 dpf (green) and 5 dpf (magenta) (N = 3). [file GLIA-70-1027-s003.tif]

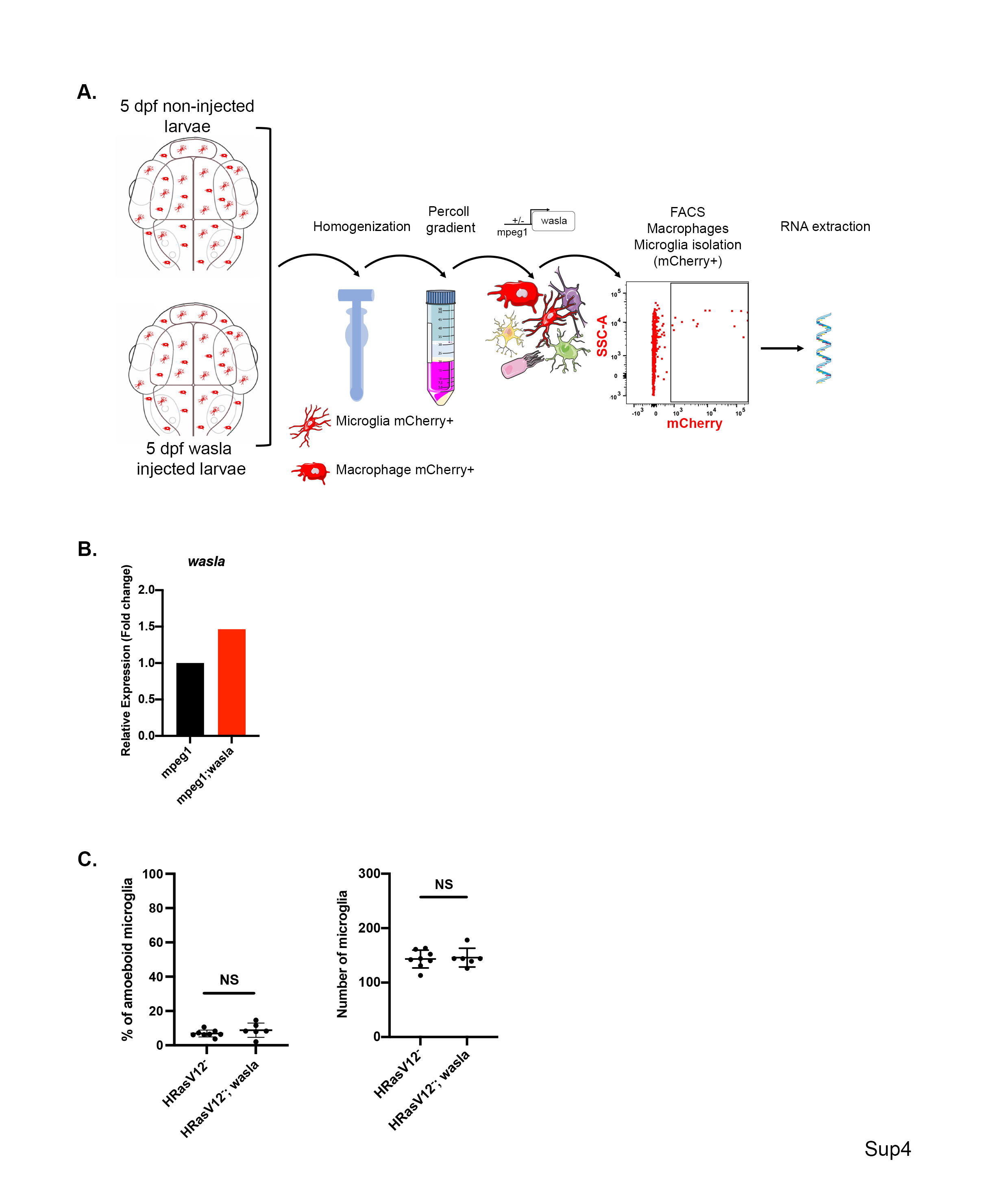

Supplement: Supplementary file 4 — Figure S4: Sup4: Walsa overexpression in microglia from HRasV12 ‐ larvae does not affect their number and morphology. (A) Schematic representation of the protocol used to isolate mCherry+ microglia/macrophages from zebrafish brains of Tg(mpeg1:mCherry) 5 dpf larvae non‐injected and injected with mpeg1:wasla plasmid to perform RNA extraction. (B) mRNA expression levels of wasla from isolated mCherry+ microglia/macrophages from 5 dpf non‐injected and injected embryos were determined by qPCR. Fold change is measured in relation to 5 dpf microglia/macrophages using the comparative (ΔΔCT) method. The means are plotted. (C) The percentage of amoeboid microglia and the total number of microglia from 5 dpf HRasV12‐ and HRasV12‐; wasla brains were quantified. HRasV12‐: n = 8; HRasV12‐; wasla: n = 6; N = 1. Error bars represent mean ± SD [file GLIA-70-1027-s004.tif]

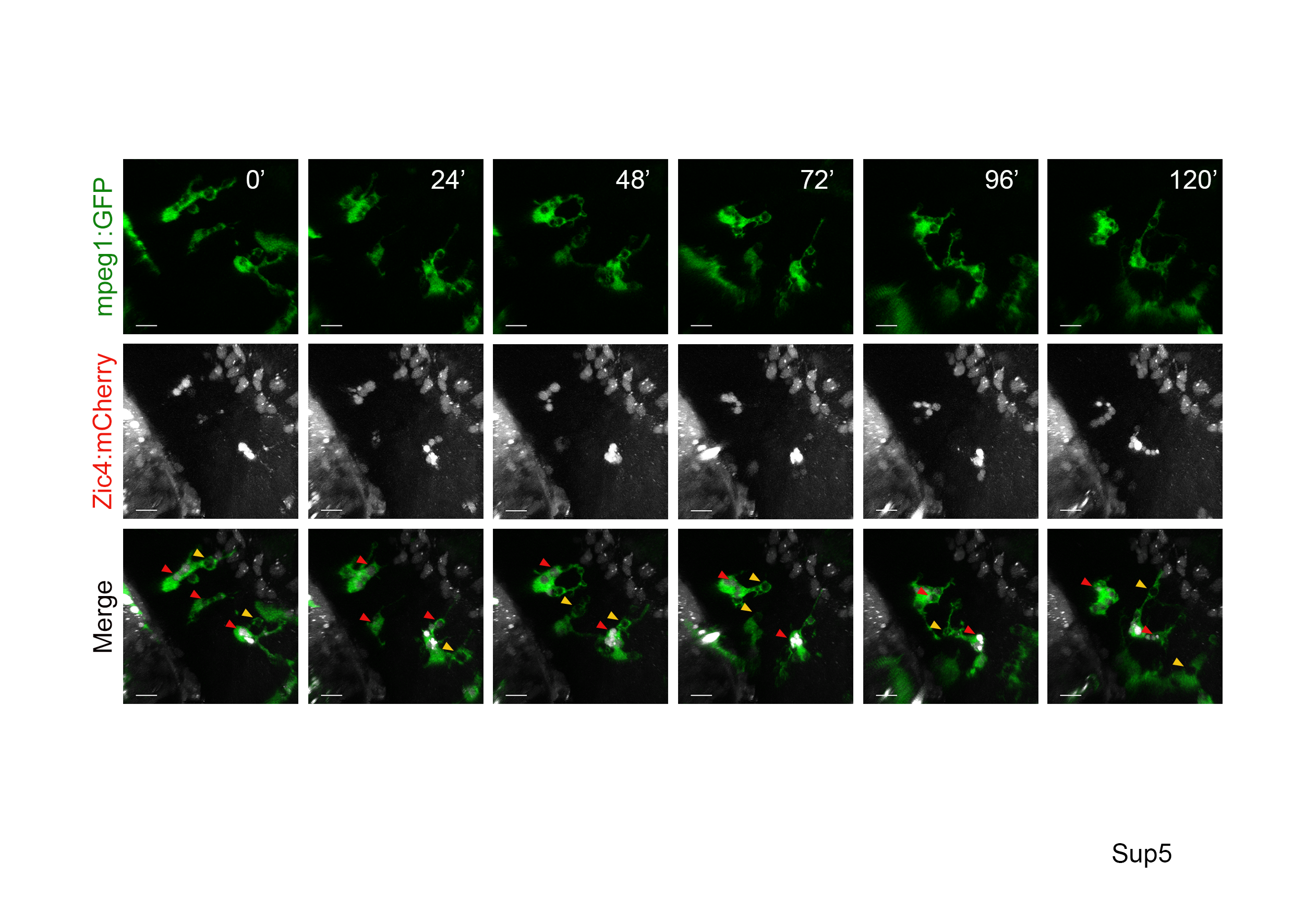

Supplement: Supplementary file 5 — Figure S5: Sup5: Phagocytosis of radial glial cell progenitors by microglia during brain development. Time‐series of movieS1, showing the clearance of radial glial cell progenitors (mCherry+) by microglia (GFP+) from 5 dpf HRasV12‐ brains. Images were captured using a Zeiss LSM880 confocal microscope with a 20X/NA 0.8 objective. Red arrowheads indicate phagosomes containing radial glial cell progenitors (mCherry+) while yellow arrowheads indicate phagosomes containing other unlabeled cell types. Scale bar represents 10 μm. All images represent the maximum intensity projections of Z stacks. [file GLIA-70-1027-s002.tif]
